# Identification and Validation of Aspartic Acid Semialdehyde Dehydrogenase as a New Anti-Mycobacterium Tuberculosis Target

**DOI:** 10.3390/ijms161023572

**Published:** 2015-09-30

**Authors:** Jianzhou Meng, Yanhui Yang, Chunling Xiao, Yan Guan, Xueqin Hao, Qi Deng, Zhongyang Lu

**Affiliations:** 1 Institute of Medicinal Biotechnology, Chinese Academy of Medical Sciences and Peking Union Medical College, Beijing 100050, China; E-Mails: mengjianzhou@126.com (J.M.); guanyan20@163.com (Y.G.); haoxueqin@hotmail.com (X.H.); dengqi1992@163.com (Q.D.); m18500395543@163.com (Z.L.); 2 The School of Basic Medicine, Ningxia Medical University, Yinchuan 750004, China; E-Mail: yyhysf@163.com

**Keywords:** aspartic acid semialdehyde dehydrogenase, *Mycobacterium tuberculosis*, pathogenicity, pristinamycin, promotor-replacement mutant, drug resistance

## Abstract

Aspartic acid semialdehyde dehydrogenase (ASADH) lies at the first branch point in the essential aspartic acid biosynthetic pathway that is found in bacteria and plants but is absent from animals. Mutations in the *asadh* gene encoding ASADH produce an inactive enzyme, which is lethal. Therefore, in this study, we investigated the hypothesis that ASADH represents a new anti-Mycobacterium tuberculosis (MTB) target. An *asadh* promoter-replacement mutant MTB, designated MTB::*asadh*, in which *asadh* gene expression is regulated by pristinamycin, was constructed to investigate the physiological functions of ASADH in the host bacteria. Bacterial growth was evaluated by monitoring OD_600_ and ASADH expression was analyzed by Western blotting. The results showed that the growth and survival of MTB::*asadh* was completely inhibited in the absence of the inducer pristinamycin. Furthermore, the growth of the mutant was rigorously dependent on the presence of the inducer in the medium. The starved mutant exhibited a marked reduction (approximately 80%) in the cell wall materials compared to the wild-type, in addition to obvious morphological differences that were apparent in scanning electron microscopy studies; however, with the addition of pristinamycin, the cell wall contents and morphology similar to those of the wild-type strain were recovered. The starved mutant also exhibited almost no pathogenicity in an *in vitro* model of infection using mouse macrophage J774A.1 cells. The mutant showed a concentration-dependent recovery of pathogenicity with the addition of the inducer. These findings implicate ASADH as a promising target for the development of novel anti-MTB drugs.

## 1. Introduction

Tuberculosis (TB), caused by *Mycobacterium tuberculosis* (MTB), remains a major threat globally, claiming millions of lives every year. Due to the prolonged persistence of MTB in the host, therapy is required for at least six months, leading to a high risk of the development of drug resistance. According to the World Health Organization (WHO) there may be 500,000 drug-resistant TB sufferers worldwide [1] and the situation is becoming much more complicated by Human Immunodeficiency Virus (HIV) co-infection [2]. The increasing prevalence of drug-resistant MTB strains has aroused increasing concern. Nevertheless, the currently available clinical anti-TB drugs, most of which were developed in the middle of last century, do not control these resistant strains. Therefore, the identification of new anti-TB drugs is an important focus of research; however, this endeavor is impeded by focusing on modifying the currently available drugs or exploiting inhibitors of known targets. Bedaquiline, which was approved by the Food and Drug Administration (FDA, USA) in 2012 for TB chemotherapy, inhibits all sensitive and resistant MTB strains by targeting ATP synthase [3], indicating that the identification of new targets represents is an effective approach to the development of new anti-infective agents. Complete sequencing of the MTB H37Rv genome makes it possible to analyze its metabolism globally [4]. The differences between human and bacterial metabolism represent an important basis of the search for new drug targets. Based on this approach, aspartic acid metabolism is a potential target. Bacteria, plants and fungi metabolize aspartic acid to produce the amino acids lysine, threonine, methionine and isoleucine in a series of reactions known as the aspartate pathway. Members of the animal kingdom do not possess this pathway and therefore, these amino acids cannot be synthesized *de novo*, but must be derived nutritionally [5]. Additionally, several important metabolic intermediates are produced by the aspartate pathway such as *meso*-diaminopimelic acid (*meso*-DAP), which is an important constituent of peptidoglycans. These molecules are essential for the maintenance of bacterial morphology and viability [6,7]. *Meso*-DAP plays a key role in peptidoglycan cross-linking by forming a covalent bond with d-alanyl or *meso*-DAP moieties of adjacent chains to generate the complete peptidoglycan [8,9].

Aspartic acid semialdehyde dehydrogenase (ASADH) is at a critical junction in the aspartate pathway, which branches at this point to produce lysine, threonine, methionine and isoleucine [10]. Therefore, we speculated that inhibiting ASADH activity will block the aspartate pathway, resulting in bacterial damage. Thus, we investigated the hypothesis that ASADH represents a new anti-MTB target.

Expression and crystallization of ASADH has allowed resolution of its structure, making it possible to identify inhibitors and study their interactions [11,12,13]. In this study, a conditional mutant *Mycobacterium tuberculosis* strain named MTB::*asadh* was constructed to study the physiologic function of *asadh*. This construct was based on the suicide plasmid pAZI9479, which contains a pristinamycin responsive protein Pip of *S. coelicolor* and the promoter (P*ptr*) of the multidrug resistance gene *ptr* of *Streptomyces pristinaespiralis* [14]. This is an excellent tool for the construction of promoter-replacement mutants, placing the inserted gene of interest under the control of P*ptr* so that its expression can be regulated strictly by the inducer pristinamycin.

The relationship between *asadh* expression and the concentration of the inducer (pristinamycin) was confirmed and then used to monitor the consequences (including viability, morphology and pathogenicity) on transformed bacteria when *asadh* was expressed at low levels. This information is required to determine the potential of ASADH as a suitable target for the development novel anti-MTB drugs.

## 2. Results

### 2.1. Construction of the Promoter-Replacement Mutant

The former fragment of *asadh* (approximately 400 bp) was amplified from the genome of *Mycobacterium tuberculosis* H37Rv, and cloned into pAZI9479 downstream of the P*ptr* to generate pAZI9479::*asadhʹ*.

**Figure 1:**
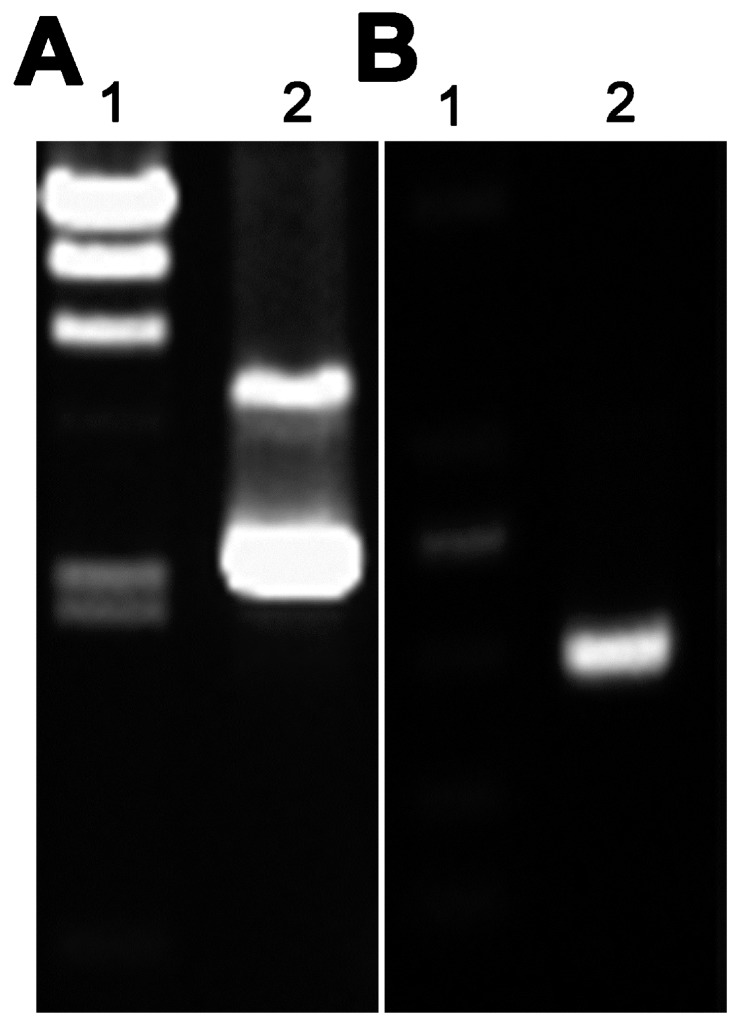
Agarose gel electrophoresis to confirm the plasmid pAZI9479 and the PCR product of *asadhʹ*. (**A**) Lane 1, λ DNA/*Hin*d III marker; Lane 2, plasmid pAZI9479; (**B**) Lane 1, DL2000 marker; Lane 2, *asadhʹ* PCR product released by diagnostic restriction enzyme (*Nco* I and *Nde* I) digestion.

The plasmid pAZI9479::*asadhʹ* was used to transform competent MTB H37Rv cells, which were then plated onto 7H10 solid medium containing 0.1 µg/mL pristinamycin and 100 µg/mL hygromycin as described previously [15]. Positively transformed bacteria formed colonies on the surface of the solid medium after incubation at 37 °C for more than four weeks. Clones were evaluated by diagnostic restriction enzyme (*Nco* I and *Nde* I) digestion to release the cloned fragment of *asadh* (approximately 400 bp), which was visualized by agarose gel electrophoresis (Figure 1). The identity of the positive mutant strain, designated MTB::*asadh*, was confirmed by sequencing and stored for later research.

### 2.2. Verification of the Essential Requirement for Asadh

**Figure 2:**
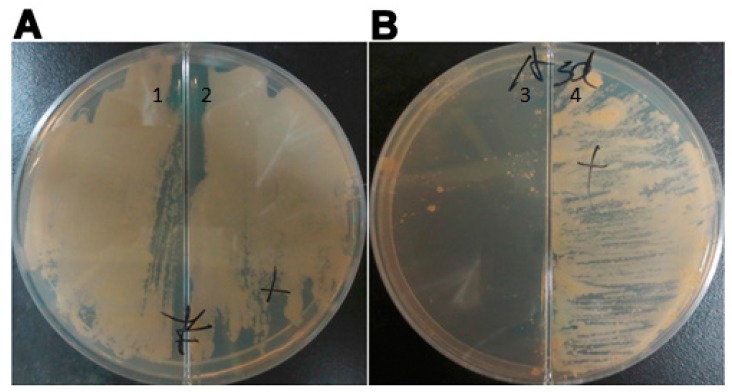
*Mycobacterium tuberculosis* cultured on 7H10 solid medium in the absence of the inducer (Section 1 and Section 3) and the presence of 0.1 µg/mL pristinamycin (Section 2 and Section 4). Plate B contained 100 µg/mL hygromycin. (**A**) MTB H37Rv bacteria formed a lawn of colonies on the medium and were dependent on the inducer for survival; (**B**) MTB::*asadh* bacteria formed colonies only on the medium containing pristinamycin. The experiments were repeated three times, and typical images are shown.

Figure 2 shows *Mycobacterium tuberculosis* cultured on 7H10 solid medium in the presence and absence of the inducer. MTB H37Rv formed a lawn of colonies on the medium in the absence of the inducer, while MTB::*asadh* formed colonies only on the medium containing pristinamycin. These observations confirmed that *asadh* was essential for MTB multiplication.

### 2.3. Growth of the Mutant

**Figure 3:**
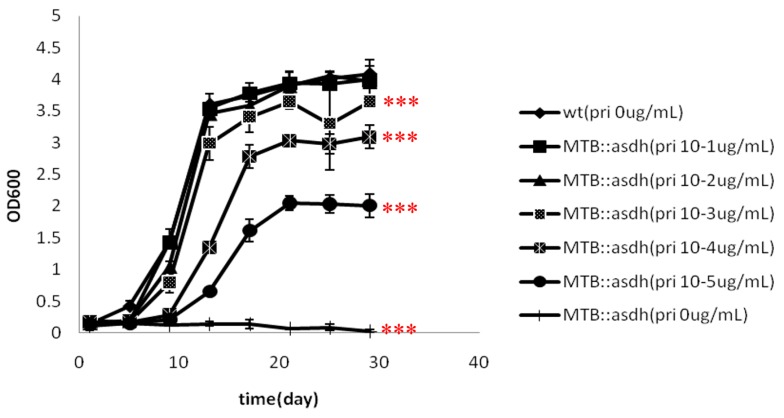
Growth of MTB and MTB::*asadh*. The wild-type (MTB) reached a plateu after two weeks in culture, reaching a maximum OD_600_ of 4.0. As the concentrations of the inducer pristinamycin decreased, the mutant grew slowed, with a reduced maximum OD_600_ of approximately 2.0. The experiments were repeated three times, and results are expressed as mean ± standard derivation (SD). Results were compared using two-way analysis of variation (ANOVA) method (*** *p* < 0.001).

To clarify that the inducer is required for growth, MTB::*asadh* bacteria were cultured in the presence of different concentrations of pristinamycin (0, 10^−5^, 10^−4^, 10^−3^, 10^−2^, 10^−1^ µg/mL). The growth curves generated by monitoring the OD_600_ values of the cultures over time are shown in Figure 3. The wild-type reached a plateau after two weeks in culture, reaching a maximum OD_600_ of 4.0. The growth of the mutant strain was strictly dependent on the presence of pristinamycin, and exhibited a concentration-dependent increase in growth over time. However, at concentrations of pristinamycin exceeding 10^−3^ µg/mL, the growth of the mutant was no longer influenced by increases in the concentration of the inducer. At concentrations of pristinamycin lower than 10^−4^ µg/mL, the growth of the mutant was markedly reduced (maximum OD_600_ was reduced by 25% (10^−4^ µg/mL) or 50% (10^−5^ µg/mL).

### 2.4. Confirmation of the Relationship between ASADH Expression and the Concentration of the Inducer

**Figure 4:**
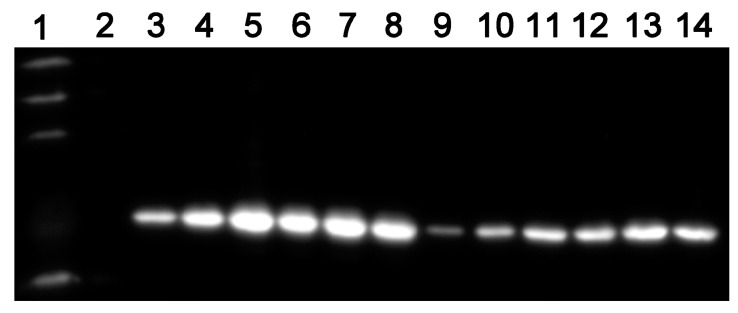
Western blot analysis of the relationship between ASADH expression and the concentration of pristinamycin over time (20 μg of total protein were loaded for cataphoresis). Lane 1, EasySee Western Marker; Lane 2 starved mutant; Lanes 3–8, ASADH expression on days 1, 3, 5, 7, 9, and 11 at 10^−1^ µg/mL; Lanes 9–14, ASADH expression on days 1, 3, 5, 7, 9, and 11 at 10^−5^ µg/mL. The experiments were repeated three times, and typical images are shown.

A shown in Figure 4, the growth of MTB::*asadh* was severely reduced (by approximately 50%) at concentrations of pristinamycin of 10^−5^ µg/mL or less, but was not affected at 10^−1^ µg/mL pristinamycin. Therefore, these two concentrations were chosen as the range over which to investigate the relationship between pristinamycin concentration and ASADH expression levels by Western blot analysis. ASADH expression was detected one day after the addition of the inducer. Its expression changed in a dose-dependent manner, with much higher ASADH expression at 10^−1^ µg/mL than at 10^−5^ µg/mL. The expression of ASADH also increased over time. Strains incubated in 7H9 medium containing pristinamycin at 10^−1^ and 10^−5^ µg/mL showed the highest expression on days 5 and 9, respectively.

### 2.5. Alterations in the Cell Wall of the Conditional Mutants

**Figure 5:**
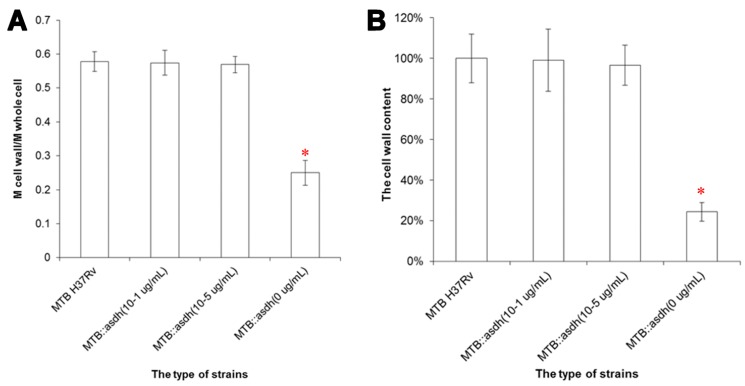
The proportion of the mass_cell-wall_ to the mass_whole-cell_. The whole cell wall was extracted from wild-type H37Rv and MTB::*asadh* bacteria cultured in the presence of 10^−1^, 10^−5^ and 0 µg/mL pristinamycin. (**A**) The proportion of the mass_cell-wall_ (M_cell-wall_) to the mass_bioplasm_ (M_bioplasm_) in the wild-type and MTB::*asadh* bacteria cultured in the presence of pristinamycin was approximately 140%, compared with approximately 30% in the MTB::*asadh* bacteria cultured in the absence of pristinamycin; (**B**) The proportion of the mass_cell-wall_ to the mass_whole-cell_ in the wild-type and MTB::*asadh* bacteria cultured in the presence pristinamycin was approximately 60%, compared with 25% in the MTB::*asadh* cells cultured in the absence of pristinamycin. The experiments were repeated three times, and results were expressed as mean ± SD. Results were compared using one-way ANOVA method (* *p* < 0.05).

ASADH is essential for the synthesis of peptidoglycans, which provide anchor points for galactan, araban and mycolic acids and are critical to the integrity of the bacterial cell wall. Therefore, we hypothesized that inhibition of elements upstream of peptidoglycan synthesis will result in disruption of the bacterial whole cell wall. To address this issue, the whole cell wall was extracted from MTB H37Rv and MTB::*asadh* bacteria in the plateau growth phase cultured in 7H9 containing 10^−1^ or 10^−5^ µg/mL pristinamycin. The proportions of the mass_cell-wall_ to the mass_whole-cell_ of these cultures are shown in Figure 5. Although the concentration of pristinamycin altered the growth rate and the maximum quantity of bacteria, the mutant cell wall contents were similar to that of the wild-type. In the absence of the inducer, the cell wall content of the starved mutant strain was reduced to 25% of the total cell weight. These data indicate that the starved mutant cells had lost an average of 77.8% of their cell wall materials.

### 2.6. Mutant Strain Pathogenicity

**Figure 6:**
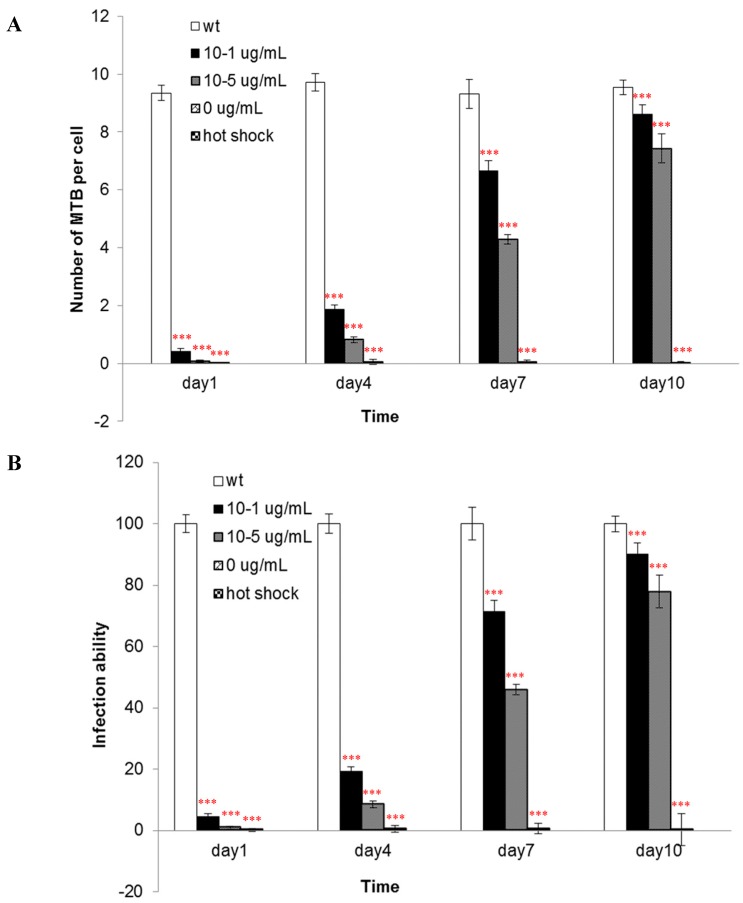
The pathogenicity of wild-type and conditional mutant MTB strains. The wild-type MTB H37Rv bacteria showed the highest infection capacity (approximately 10 bacteria per macrophage) (**A**), while the capacity of the mutant to infect macrophages was gradually increased with the concentration of pristinamycin and the period of induction (**B**). The experiments were repeated for three times, and results were expressed as mean ± SD. Results were compared using one-way ANOVA method (*** *p* < 0.001).

The integrity of the cell wall is important for the pathogenicity of MTB. In this study the pathogenicity of the conditional mutant strain was evaluated by investigation of the ability to infect the mouse macrophage cell line, J774A.1. Figure 6 shows that the amount of the mutants invading into macrophages was gradually increased with the period of induction.

### 2.7. Recovery of Cell Morphology

**Figure 7:**
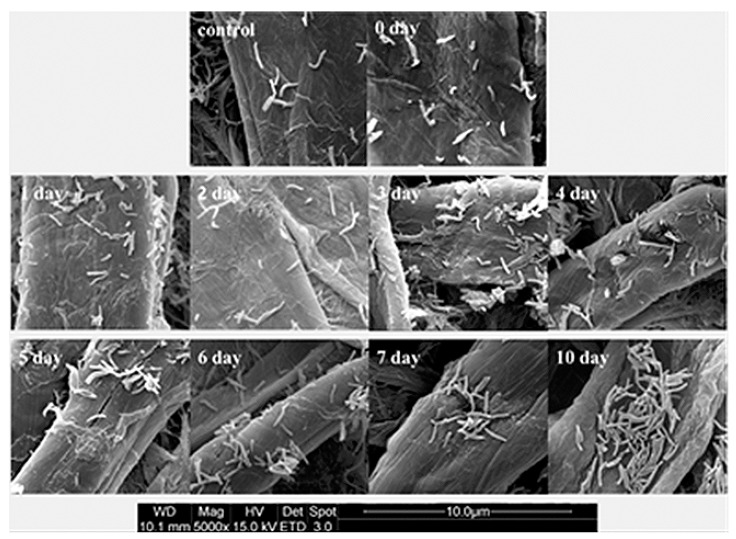
Morphological changes in the conditional mutant MTB strain. Wild-type MTB H37Rv bacteria appear as long rods of uniform size, while the starved mutant MTB bacteria are spherical and irregular in size. The mutant MTB bacteria gradually elongated with the period of incubation with the inducer pristinamycin, until the rod-shaped morphology similar to that of the wild-type MTB was recovered.

The cell wall is the primary element responsible for the maintenance of morphology. The effect of the inducer on the morphology of starved mutant cells was investigated by scanning electron microscopy (Figure 7). Wild-type MTB H37Rv bacteria in the logarithmic phase of growth appeared as long rods of uniform size, while the starved mutant MTB bacteria were spherical and irregular in size. However, the shape of the mutant MTB bacteria gradually elongated with the period of incubation with the inducer pristinamycin, until rod-shaped morphology similar to that of the wild-type MTB was recovered.

## 3. Discussion

The differences between human and bacterial metabolism have been highlighted as a new approach to the identification of novel antibacterial targets. The aspartate pathway exists in plants, fungi, archaea and microbes, but not in animal; therefore, the enzymes that catalyze the reactions involved in this pathway are implicated as potential antibacterial targets. A previous knockout study showed that aspartate kinase (ASK, EC 2.7.2.4, Rv3709c), the first enzyme in the aspartate pathway, is essential for the growth and survival of *Mycobacterium smegmatis* [16]. However, to date, there are no reports describing the anti-MTB effects of targeting ASADH, which catalyzes the second step in the aspartate pathway. Therefore, in this study, we investigated the hypothesis that ASADH represents a new anti-*Mycobacterium* *tuberculosis* (MTB) target by constructing a conditional mutant in which expression of the *asadh* gene is regulated by pristinamycin.

The conditional mutant strain, MTB::*asadh*, was successfully generated and the expression of ASADH was shown to be induced by pristinamycin in a time- and dose-dependent manner in the range of concentrations from 10^−5^ to 10^−2^ µg/mL. Furthermore, MTB::*asadh* did not grow in the absence of the inducer (Figure 2); thus, demonstrating that *asadh* is essential to the growth of MTB.

The absence of *asadh* expression results in an inadequate supply of the downstream products of the aspartate pathway. Lysine, threonine, methionine and isoleucine are required for bacterial growth. Our results showed that the growth of MTB::*asadh* was increased with the concentration of the inducer (Figure 3). It can be speculated that the induction of ASADH expression with increased pristinamycin concentrations (Figure 4) resulted in a concentration-dependent recovery of the production of lysine, threonine, methionine and isoleucine, with a concomitant recovery in bacterial growth.

*Meso*-DAP, an intermediate of the aspartate pathway, is required for cell wall synthesis. We showed that the proportion of cell wall material in MTB::*asadh* was reduced by approximately 80%, compared to the wild-type MTB, but was restored in the presence of the inducer, thus confirming that *asadh* expression is essential for the generation of the bacterial cell wall (Figure 5). In any case, the wild-type MTB was cultured in limited 7H9 medium without carbon source (such as glycerol and dextrose) for almost two months, and its cell wall contents (55.4% ± 2.7%) revealed no statistical difference with the bacteria cultured in normal 7H9 medium (*p* > 0.05, *t*-test). Further, the cell wall contents of MTB cultured in 7H9 medium with sorbitol and 32 µg/mL D-cycloserine (DCS) for two weeks was only 24.5%, which was similar to that of the starved mutants (*p* > 0.05, *t*-test); however, it had significant statistical difference with those grown without DCS (*** *p* < 0.005, *t*-test), while the bacteria cultured in 7H9 medium with sorbitol almost had no influence on their cell wall contents (*p* > 0.05, *t*-test) (data not shown). These results illustrate that the cell wall would be damaged when *asadh* expression shuts down.

The components of the cell wall are important for the infectivity and virulence of MTB, also our results indicated that the mutants’ capacity to enter into macrophages was attenuated when *asadh* was down-regulated and their infectivity were restored as the *asadh* expressed (Figure 6). Moreover we investigated the effects of the absence of *asadh* on the bacterial cell morphology. Compared with the uniform rod-shaped morphology of the wild-type strain, MTB::*asadh* exhibited altered morphology appearing as irregularly-sized spheroids (Figure 7). This is likely to be the result of the loss of cell wall components as a result of the absence of ASADH. With the addition of the inducer pristinamycin, the proportion of the cell wall material and morphology of the mutant returned to that of the wild-type strain (Figure 5 and Figure 7).

The results described clarify that the *asadh* gene is essential in order for MTB to maintain normal growth, pathogenicity and morphologyWe therefore conclude that ASDH may be a potential anti-TB target. ASDH should be expressed heterogenously in *E. coli* for obtaining substantial pure enzyme to establish a high-throughput model to screen for its inhibitors which could be developed as new anti-TB drugs.

## 4. Experimental Section

### 4.1. Bacteria

All chemicals used were purchased from Sigma unless otherwise stated. *Mycobacterium tuberculosis* H37Rv (*ATCC*27294) was cultured in Middlebrook 7H9 broth (supplemented with glycerol and polysorbate 80) in combination with Middlebrook ADC enrichment or 7H10 agar solid media supplemented with OADC (ADC + 0.003% oleic acid) [17]. *Escherichia coli* DH5α (TransGen Biotech, Beijing, China) were cultured in Luria–Bertani (LB) broth or on LB agar medium. Hygromycin (Amresco, Anachem, Bedfordshire, UK) was added if necessary at 200 µg/mL for *Escherichia coli* and 100 µg/mL for *Mycobacterium*. All chemicals were purchased from Amresco unless otherwise described.

### 4.2. Molecular Manipulation

Plasmid pAZI9479 was the kindly gift of Professor Francesca Forti (Dipartimento di Scienze Biomolecolari e Biotecnologie, Università degli Studi di Milano, Via Celoria, Milano, Italy) [14]. All PCR reagents were purchased from TransGen Biotech. Genomic DNA was extracted from MTB H37Rv in the logarithmic phase for use as the template in PCRs according to previously described protocols [18]. Primers were designed for amplification of the former fragment of the *asadh* gene using the Primer Premier 5.0 software based on the sequence published in the NCBI database (GenBank accession number: 885118) as follows: *asadh′* F (5′-AAAACCATGGTGCATCATCATCATCATCACGGCCTGTCAATAGGGA) and *asadh′* R (GCGGCATATGTGGTGCAGTTCGGGTTGGCGA). The shaded box indicates the restriction enzyme digestion sites (*Nco* I and *Nde* I) introduced for the purposes of directional cloning and the underlined bases indicate the six-histidine-tag used to detect the protein expression in the mutant bacteria at different concentrations of the inducer.

The PCR conditions were as follows: hot start at 94 °C for 3 min, followed by 30 cycles of 94 °C for 45 s, 58 °C for 50 s, and 72 °C for 30 s, and a final extension at 72 °C for 10 min. The purified PCR product was digested with the *Nco* I and *Nde* I (TaKaRa, Shiga, Japan) and then ligated into the corresponding sites of plasmid pAZI9479 using T4 ligase (TaKaRa). The ligation mixture was used to transform *Escherichia coli* DH5α competent cells and subclones were sequenced to identify the positive strains containing pAZI9479::*asadh′*.

### 4.3. Construction and Characterization of Conditional Mutants

The bacteria cell cultures and plasmid purification were performed as previously described [19,20,21]. Briefly, 0.1 volumes of 2 M glycine was added to the culture 48 h before collection of the logarithmic phase cells. These bacteria were washed three times with 10% glycerol, reducing the volume each time. Finally the bacteria were suspended in 1/500 volumes of ice-cold 10% glycerol for later use. The plasmid pAZI9479::*asadh′* was pretreated with 100 mJ/cm^2^ UV irradiation in a Spectrolinker™ UV crosslinker (Spectronics, Upland, CA, USA). No more than 5 µL treated DNA was mixed with 200 µL competent cells and the mixture was then transferred to a 0.2 cm cuvette (Bio-rad, Hercules, CA, USA) for electroporation according to the following program: voltage 2.5 kV, capacitance 25 µF, resistance 1000 Ω, and a pulse time of 15–25 ms. The bacteria were incubated in 5 mL 7H9 broth for 24–48 h at 37 °C to recover viability before being plated onto 7H10 solid medium containing 0.5 µg/mL pristinamycin (Santa Cruz Biotechnology, Inco., Santa Cruz, CA, USA) and 100 µg/mL hygromycin. The plates were then incubated at 37 °C for 4 weeks, before the colonies formed were selected for sequencing to confirm replacement of the *asadh* promoter with the *ptr* promoter (P*ptr*). The primers were P*ptr* F (GATCACCGCCTGGGTCCAGGACGA), the upstream fragment of P*ptr* and *asadh* R (CACAAGTCGGCGGTCAGC), the downstream complete *asadh* fragment.

### 4.4. Verification of the Essential Requirement for Asadh

To confirm the requirement for *asadh* expression, 7H10 solid medium containing 100 µg/mL hygromycin was poured into a plate containing a divider to separate the solid medium supplemented with or without the inducer pristinamycin AI. The mutant strain was spread onto the surface and incubated at 37 °C for 4 weeks until colonies were confirmed to form only on the medium containing the inducer.

### 4.5. Growth of the Conditional Mutant

The mutant strains were washed three times with PBST buffer (8 mM NaCl, 2.6 mM KCl, 1.4 mM K_2_HPO_4_, 8 mM Na_2_HPO_4_, and 0.05% (*w*/*v*) Tween 80, pH 7.4) to remove the inducer. The bacteria were cultured in broth containing no inducer but containing 500 mM sorbitol at 37 °C for approximately 2 months to deplete the endogenous ASADH; this was defined as starvation. The growth of starved bacteria were cultured in the presence of different concentrations of the inducer (0, 10^−5^, 10^−4^, 10^−3^, 10^−2^, 10^−1^ µg/mL pristinamycin) was estimated by monitoring OD_600_ and the generation of growth curves.

### 4.6. ASADH Expression of the Conditional Mutants

To clarify the relationship of the ASADH expression levels with the inducer concentrations, the starved mutant cultured in the presence of different concentrations of pristinamycin (0, 10^−5^, 10^−1^ µg/m) and sampled (3 mL) every two days for ten days. ASADH expression was analyzed by Western blot detection of the His-tag as described previously [22]. The EasySee Western Marker was purchased from TransGen Biotech.

### 4.7. Alterations in the Cell Wall of the Conditional Mutants

Starved mutant bacteria were cultured in 7H9 medium in the absence or presence of pristinamycin (10^−1^ or 10^−5^ µg/mL) for 2 weeks. The bacterial cell wall was extracted as described by Besra [23]. Briefly, bacteria at exponential growth phase were re-suspended in lysis buffer containing 2% *w*/*v* Triton X-100 in PBS (0.1 M K_2_HPO_4_, 0.01 M NaCl, pH 7.4) at a density of 0.5 g bacteria (wet weight) per 1 mL buffer, and then sonicated on ice, followed by centrifugation at 27,000× *g* at 4 °C for 15 min. The insoluble sediment was further extracted in the lysis buffer overnight. Thereafter, the insoluble sediment was washed with lysis buffer, and extracted with PBS containing 2% SDS at 95 °C for 1 h to remove the associated proteins. The pellet was then washed with distilled water, 80% acetone in water, and acetone successively. The purified cell wall was lyophilized. All these experiments were carried out in triplicate, and the data was expressed as mean ± standard deviation.

### 4.8. Pathogenicity of the Mutants

The murine macrophage cell line J774A.1 (American TypeCulture Collection, ATCC (Rockefeller, Maryland, USA)) were cultured in DMEM medium (Gibco, Carlsbad, CA, USA) containing 10% (*v*/*v*) fetal calf serum. The macrophage infection procedures were according to Zhang, J. [24]. Starved mutant bacteria were cultured in 7H9 medium containing different concentrations of pristinamycin (0, 10^−5^, 10^−1^ µg/mL). Wild-type bacteria were used as a control. After 1, 4, 7 and 10 days in culture, bacteria were collected and washed three times with PBS buffer. The bacteria were suspended in DMEM and the final OD600 value was adjusted to 0.5 (the corresponding cell number was about 0.5 × 10^6^/mL). One day before infection, three days cultured macrophage cells were washed with PBS, suspended in DMEM medium, and the cells were diluted to 5 × 10^4^ cells per milliliter. DMEM medium mixed with equal volume (0.5 mL) macrophage cells were incubated in 24-well plates for 12 h to allow cell adherance to plates. The adhered cells were washed with PBS buffer, and 0.5 milliliter quantified bacteria were added to the 24-well plates. So the multiplicity of infection (MOI) was 10:1. These mixtures were incubated for 2 h for infection and amikacin was added with final concentration of 200 µg/mL to kill bacteria which did not invade into cells. 2 h later the plates were washed three times with PBST buffer, and then macrophage cells were lysed with PBS buffer containing 2% Triton X-100. The lysates were spread on 7H10 solid broth in a serial dilutions, and the colonies were counted after two weeks incubation at 37 °C.

### 4.9. Morphology of the Mutants

Starved mutant bacteria were cultured in 7H9 medium containing 10^−1^ µg/mL pristinamycin and 100 µg/mL hygromycin. Cultures were sampled (2 mL) every day for ten days and bacterial morphology was evaluated by electron microscopy as described by Tahlan [15]. Wild-type *Mycobacterium tuberculosis* H37Rv in log phase growth was used as a positive control and the starved mutant strain was used as a negative control.

### 4.10. Statistical Analysis

The experiments were repeated three times. The program used to process these data was EXCEL (Microsoft, Redmond, Redmond, Washington, DC, USA), and the statistics method was AVERAGE+/−STDEV. Results between different groups were compared using two-way or one-way analysis of variation (ANOVA) method.

## 5. Conclusions

We further investigated pathogenicity of the MTB::*asadh* strain in an *in vitro* model of infection using the mouse macrophage cell line, J774A.1. The MTB::*asadh* strain was unable to infect J774A.1 macrophages; however, the infection ability of the mutant gradually increased with the concentration of pristinamycin and the period of induction. These observations suggest that the integrity of the cell wall is critical for bacterial infectivity. Thus, it can be concluded that inhibition of the bacterial aspartate pathway by disrupted expression of ASADH results in abrogation of the infectious capacity of MTB.

The results of this study indicate that ASADH represents a promising target for the development of novel anti-MTB drugs and that specific inhibitors may successfully bypass existing drug resistance mechanisms that threaten the control of TB.
